# The GG Genotype of Telomerase Reverse Transcriptase at Genetic Locus rs2736100 Is Associated with Human Atherosclerosis Risk in the Han Chinese Population

**DOI:** 10.1371/journal.pone.0085719

**Published:** 2014-01-21

**Authors:** Lei Feng, Shi-yan Nian, Jihong Zhang

**Affiliations:** 1 Department of Laboratory, People's Hospital of Yuxi City, Yuxi, Yunnan Province, China; 2 Intensive Care Unit, People's Hospital of Yuxi City, Yuxi, Yunnan Province, China; 3 Faculty of Medicine, Kunming University of Science and Technology, Kunming, Yunnan Province, China; National Institute for Viral Disease Control and Prevention, CDC, China, China

## Abstract

A single nucleotide polymorphism (SNP) in the second intron of human TERT (hTERT), rs2736100, acts as a critical factor in hTERT synthesis and activation. The rs2736100 SNP was found to be associated with susceptibility to many cancers. Recently, inhibition of telomerase and marked telomere shortening were determined to be closely associated with the increasing severity of atherosclerosis. The association between the SNP of rs2736100 and the presence of atherosclerosis was evaluated in 84 atherosclerosis patients and 257 healthy controls using multivariate logistic regression analyses. The proportion of the GG genotype in atherosclerosis patients (17.9%) was significantly higher than in the control group (9.7%). Eight variables, including age, gender, cholesterol, high density lipoprotein, homocysteine, total bilirubin, indirect bilirubin, and rs2736100 GG genotype, were associated with atherosclerosis with odds ratios of 1.88, 2.11, 1.66, 0.23, 1.27, 1.29, 1.53, and 1.74, respectively, using multivariate logistic regression analyses. Homozygous GG was demonstrated to be associated with the presence of atherosclerosis in our population.

## Introduction

A telomere is a region of repetitive nucleotide sequences at the end of each chromatid of most eukaryotic organisms that protects the end of the chromosome from deterioration or from fusing with neighboring chromosomes [1]. Telomere length varies greatly between species, from approximately hundreds of base pairs in yeast to many kilobases in humans, and usually is composed of arrays of guanine-rich, six- to eight-base-pair repeats [2], [3]. Without telomeres, genomes would progressively lose information and be truncated after cell division because enzymes that duplicate DNA cannot continue their duplication all the way to the end of chromosomes [1]–[3]. Therefore, telomeres act as a disposable buffer zone at each end of the chromosomes [4]. Nevertheless, telomeres will be consumed during cell division [1]–[4]. Telomerase reverse transcriptase (TERT) is a catalytic subunit of the telomerase, which together with the telomerase RNA component (TERC), are the most important components of the telomerase complex [5]. Telomerase is a ribonucleoprotein polymerase that maintains telomere ends by addition of the telomere repeat TTAGGG [6]. The enzyme consists of a protein component with reverse transcriptase activity, encoded by the gene, and an RNA component that serves as a template for the telomere repeat [5], [6]. Telomerase expression plays a role in cellular senescence because it is normally repressed in postnatal somatic cells, resulting in progressive shortening of telomeres [5], [6].

The telomere length of a species is determined by the heredity and frequency of cell division [7]. There are theories that claim that steady shortening of telomeres with each replication in somatic cells may have a role in senescence and in the prevention of cancer [7]. Chronic inflammation could accelerate the telomere to shorten via increased cell division frequency, and oxidative stress could directly result in telomere deletion [8].

Human TERT (hTERT) is located in 5p15.33 [9], and rs2736100 is located in the second intron of hTERT. Reports have shown that rs2736100 acts as a critical factor in hTERT synthesis, in charge of hTERT activation [10]. A single nucleotide polymorphism (SNP) of rs2736100 was identified to be associated with susceptibility to idiopathic pulmonary fibrosis [11], lung cancer [12], [13], glioma [14], testicular germ cell cancer, and bladder cancer [15], [16].

Atherosclerosis is the principal cause of myocardial infarction, stroke, and peripheral vascular disease, and accounts for nearly half of all mortality in developed countries [17]–[19]. Atherosclerosis is the buildup of inflammatory cells (macrophages and T lymphocytes), vascular smooth muscle cells (VSMCs), and intracellular and extracellular lipids on the inner walls of arteries [17]–[19]. These plaques can restrict blood flow to the heart muscle by physically clogging the artery or by causing abnormal artery tone and function [17]–[19]. Abnormal VSMC proliferation is thought to contribute to the pathogenesis of vascular occlusive lesions, including atherosclerosis [20]. Vigorous division and remodeling of VSMCs might consume telomeres. Recently, inhibition of telomerase and marked telomere shortening were found to be closely associated with the increasing severity of atherosclerosis [21]. Because hTERT is the key molecular complex that maintains telomere stabilization, genetic polymorphisms in hTERT might be associated with atherosclerosis. In this report, the relationship between a SNP of rs2736100 and atherosclerosis was evaluated in a case-control study.

## Materials and Methods

### Patients

To assess genetic polymorphisms related to atherosclerosis, we recruited 84 ethnic Han Chinese patients (66 males and 18 females; age range, 36–79 years; median age, 62 years) with atherosclerosis who were unrelated consecutive inpatients at the People's Hospital of Yuxi City, China between November 2010 and November 2012. To obtain an estimate of the genetic distribution of the reference allele in the general population in China, we also randomly obtained DNA samples from 257 healthy individuals who visited People's Hospital of Yuxi City (153 men and 104 women; age range, 18–58 years; median age, 34 years) with no history of atherosclerosis. The review board of People's Hospital of Yuxi City approved this study (approval number: YNYXH2010-003). Written informed consent was obtained according to the guidance of the Chinese National Ethics Regulation Committee. Participants were simultaneously informed of their right to repeal consent by themselves or their kin, caretakers, or guardians.

Patients were diagnosed with atherosclerosis according to American Heart Association guidelines [22]. Individuals with high blood pressure (Hypertension is defined as systolic pressure greater than 140 mm Hg or diastolic pressure greater than 90 mm Hg.), alcohol abuse, diabetes, and smoking history were rejected from the study group. All 84 atherosclerosis patients were further confirmed using radionuclide angiography. The 257 healthy controls did not have a history of chronic diseases, autoimmune diseases, and any cardiovascular diseases. The following clinical parameters were obtained for each patient at the time of whole-blood collection: age, gender, triglycerides, cholesterol, high density lipoprotein, low density lipoprotein, apolipoprotein A1, apolipoprotein B, lipoprotein (a), uric acid, homocysteine, total bilirubin, γ-glutamyltransferase, and indirect bilirubin.

### Polymorphism genotyping

Genomic DNA was extracted from 100 µl of whole blood using the QIAamp DNA Blood Mini Kit (Gaithersburg, MD) according to the manufacturer's instructions. Extracted DNA was dissolved in 20 µl of 10 mM Tris-HCl buffer (pH 8.0) containing 1 mM EDTA, and DNA samples were stored at −30°C until use.

**Figure 1:**
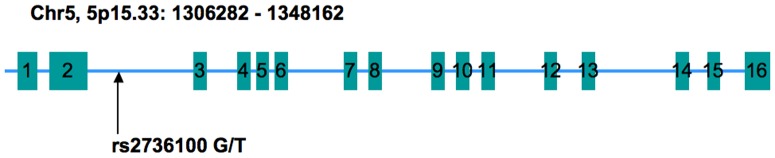
Location of rs2736100 in the hTERT Gene. Exon and intron organization of the hTERT gene, including the location of rs2736100. Blue rectangles indicate the 16 exons, spaces between two exons were introns.

Genetic polymorphisms in hTERT were determined by MassARRAY®System (San Diego, CA) according to the manufacturer's user guide. We used the primers F (5′-ACGTTGGATGTGACACCCCCACAAGCTAAG-3′) and R (5′- ACGTTGGATGACAAAGGAGGAAAAGCAGGG-3′) to amplify the specific hTERT fragment that covers rs2736100 (Figure 1). A 90-bp hTERT fragment was PCR amplified with extracted genomic DNA as the template. PCR was performed using HiFifast DNA polymerase (Biovisualab Inc, Shanghai, China). The thermocycling conditions were as follows: 94°C for 10 min, followed by 35 cycles of 94°C for 30 s, 57°C for 60 s, and 72°C for 60 s. To verify the size of the PCR product, amplicons were visualized on 12.5% polyacrylamide gels with appropriate size markers.

The MassARRAY system is based on single-base primer extension technology. The MassARRAY technology uses matrix-assisted laser desorption ionization time-of-flight (MALDI-TOF) mass spectrometry to measure directly the mass of the extension product(s) and then correlates the detected mass with a specific genotype. The extension primer sequence is 5′-TCCGTGTTGAGTGTTTCT-3′, and for details of the protocol, please refer to SNP Genotyping Using the Sequenom MassARRAY iPLEX Platform.

### Statistical analyses

We used a multivariate logistic regression model to calculate the statistical power required to detect the contribution of a SNP to atherosclerosis risk while including other known risk factors for atherosclerosis. SNP status was assigned as X = 0, 1, or 2, which represent homozygous for an allele, heterozygous, or homozygous for a different allele, respectively. The required sample size, n, for the multivariate logistic regression analysis was calculated using the following formula [23], [24]:
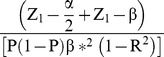
where Z_u_ is the upper *u*th percentile of the standard normal distribution; *P* is the proportion of patients with atherosclerosis when X = 1; β* is the size of the effect of an SNP; and *R* ^2^ is the multiple correlation coefficient (we used 0.009 based on our data) relating X with the following covariates: age, male gender, cholesterol, high density lipoprotein, homocysteine, total bilirubin and indirect bilirubin [25]. When the effect size of a SNP was assumed to be 0.69, which corresponded to an odds ratio (OR) of 2, the required sample size was calculated to be 75 or 100 for a statistical power of 80% or 90%, respectively. Based on these calculations, our sample size was sufficient for conditions in which the OR of an SNP exceeded 2.

Associations between the clinical parameters (age, gender, triglycerides, cholesterol, high density lipoprotein, low density lipoprotein, apolipoprotein A1, apolipoprotein B, lipoprotein (a), uric acid, homocysteine, total bilirubin, γ-glutamyltransferase, indirect bilirubin, and rs2736100 genotype) and the presence of atherosclerosis were evaluated using Student's t-tests, Mann–Whitney U tests, and χ^2^ tests. Associations between the genotype of each locus and the presence of atherosclerosis were evaluated using χ^2^ tests. The Cochran–Armitage test was used to test for trends. Possible confounding effects among these variables were adjusted using a multivariate logistic regression model, and the ORs and 95% confidence intervals (CIs) were calculated. *P*<0.05 was considered significant in the two-tailed tests. The Hardy–Weinberg equilibria of alleles at individual locus were evaluated using the HWE program.3 (ftp://linkage.rockefeller.edu/software).

## Results

### Patients' characteristics

Of the 84 atherosclerosis patients, 66 were male and 18 were female, with an age range from 36 to 79 years and a median age of 62 years. Of the 257 healthy individuals, 153 were male and 104 were female, with an age range from 18 to 58 years and a median age of 34 years. As shown in Table 1, there were no significant differences in triglycerides, apolipoprotein A1, or direct bilirubin levels between the groups with and without atherosclerosis. In the atherosclerosis group, cholesterol, low-density lipoprotein, apolipoprotein B, lipoprotein (a), uric acid, homocysteine, total bilirubin, γ-glutamyltransferase, and indirect bilirubin levels were higher, whereas high-density lipoprotein was lower in the control group, which is consistent with current reports [22], [25].

**Table 1:** Clinical biochemical indexes associated with atherosclerosis.

Variable	Total (n = 341)	Case (n = 84)	Control (n = 257)	*P*
Age	40 (18–79)	62 (36–79)	34 (18–58)	0.015
Gender, male (female)	219 (122)	66 (18)	153 (104)	0.002
Triglycerdes (mmol/L)	2.18±1.60	1.93±1.55	2.26±1.62	0.096
Cholesterol (mmol/L)	4.41±1.06	4.84±1.29	4.26±0.93	<0.001
High density lipoprotein (mmol/L)	1.08±0.28	1.17±0.27	1.04±0.28	0.001
Low density lipoprotein (mmol/L)	2.38±0.73	2.63±0.86	2.30±0.66	0.002
Apolipoprotein A1 (mmol/L)	1.23±0.24	1.25±0.26	1.22±0.23	0.43
Apolipoprotein B (mmol/L)	0.69±0.21	0.83±0.25	0.64±0.18	<0.001
Lipoprotein (a) (mg/L)	163±164	246±192	135±144	<0.001
Uric acid (umol/L)	333±92	370±85	321±91	<0.001
Homocysteine (umol/L)	15.78±6.36	21.20±7.68	14.01±4.68	<0.001
Total bilirubin (umol/L)	13.20±5.72	9.75±3.32	14.33±5.90	<0.001
Direct bilirubin (umol/L)	4.03±1.59	3.87±1.18	4.08±1.70	0.219
γ-glutamyltransferase (U/L)	33.31±24.88	44.69±35.94	29.6±18.63	<0.001
Indirect bilirubin (umol/L)	9.17±4.45	5.88±2.48	10.25±4.42	<0.001

Normally distributed data were presented as means ± standard deviation (SD), skewed data were presented as the median (interquartile range). Clinical parameters were evaluated to determine an association with the presence of atherosclerosis using the t test or the Mann–Whitney U test.

### rs2736100 allele distribution

The Hardy–Weinberg equilibria of alleles were evaluated using the HWE software (ftp://linkage.rockefeller.edu/software). The allele frequency in the atherosclerosis and control groups was in Hardy-Weinberg equilibrium (*P*>0.05), suggesting that allele frequencies in the case and control populations remain constant in their genetic background. The proportion of the GG, GT, and TT genotypes in atherosclerosis patients were 17.9%, 57.1% and 25.0%, respectively. The proportion of the GG, GT, and TT genotypes in control group were 9.7%, 66.5% and 23.7%, respectively. The proportion of the GG genotype in atherosclerosis patients (17.9%) was significantly higher than in the control group (9.7%) (Table 2). There was no significant difference in the G allele frequency between atherosclerosis patients (46.4%) and controls (43.0%), suggesting that only homozygous GG was associated with atherosclerosis.

**Table 2:** The frequence of rs2736100 alleles in case and control cohort.

Genotypes	Case (n = 84)	Control (n = 257)
GG	15 (17.9)	25 (9.7)
GT	48 (57.1)	171 (66.5)
TT	21 (25.0)	61 (23.7)

Proportion of rs2736100 alleles are shown as frequency (percentage).

### Associations of rs2736100 with atherosclerosis

The rs2736100 GG homozygote (*P* = 0.041), cholesterol ≥4.4 mmol/L (*P*<0.001), low-density lipoprotein ≥2.38 mmol/L (*P* = 0.002), apolipoprotein B ≥0.69 mmol/L (*P*<0.001), lipoprotein (a) ≥163 mg/L (*P*<0.001), uric acid ≥333 µmol/L (*P*<0.001), homocysteine ≥15.78 µmol/L (*P*<0.001), total bilirubin ≥13.20 µmol/L (*P*<0.001), γ-glutamyltransferase ≥33.31 U/L (*P*<0.001), indirect bilirubin level ≥9.17 µmol/L (*P*<0.001), and high-density lipoprotein ≤1.08 mmol/L (*P* = 0.001) were significantly associated with the presence of atherosclerosis by Student's t-tests, Mann–Whitney U tests, and χ2. To eliminate any possible multicollinearity between or among variables, collinearity diagnostics were performed before further statistical analysis. cholesterol, low-density lipoprotein and apolipoprotein B displayed collinearity, thus only cholesterol was included in following multivariate logistic regression analyses to avoid multicollinearity among cholesterol, low-density lipoprotein and apolipoprotein B.

To further evaluate the effect of the rs2736100 GG genotype on atherosclerosis, multivariate logistic regression analyses were performed using these variables (rs2736100, age, geder, cholesterol, uric acid, homocysteine, total bilirubin, γ-glutamyltransferase, indirect bilirubin, and high-density lipoprotein). Eight variables, including age, gender, cholesterol, high density lipoprotein, homocysteine, total bilirubin, indirect bilirubin, and rs2736100 GG genotype, were included in the final model with odds ratios of 1.88 (≥50 yrs vs. <50 yrs), 2.11 (male vs. <female), 1.66 (≥4.4 mmol/L vs. <4.4 mmol/L), 0.23 (≥1.08 mmol/L vs. <1.08 mmol/L), 1.27 (≥15.78 µmol/L vs. <15.78 µmol/L), 1.29 (≥13.20 µmol/L vs. <13.20 µmol/L), 1.53 (≥9.17 µmol/L vs. <9.17 µmol/L) and 1.74 (GG vs. TT) (Table 3), respectively.

**Table 3:** Factors associated with presence of atherosclerosis in multivariate analysis.

Independent variables	Odd ratio	95% CI	*P* value
rs2736100 (GG vs.TT)	1.74	1.15–4.63	0.021
Age	1.88	1.32–4.23	0.032
Gender	2.11	1.01–4.55	0.031
High density lipoprotein (≥1.08 mmol/L vs. <1.08 mmol/L)	0.23	0.07–0.66	0.001
Homocysteine (≥15.78 umol/L vs. <15.78 umol/L)	1.27	1.12–3.31	0.031
Total bilirubin (≥13.20 umol/L vs. <13.20 umol/L)	1.29	1.22–3.43	0.020
Indirect bilirubin (≥9.17 umol/L vs. <9.17 umol/L)	1.53	1.20–3.63	0.039
Cholesterol (≥4.4 mmol/L vs. <4.4 mmol/L)	1.66	1.13–4.65	0.032

95% CI, 95% confidence interval.

## Discussion

Reports have shown that rs2736100 acts as a critical factor in hTERT synthesis [1]–[3]. Normally, telomerase activity in somatic cells is silent or low, whereas in tumor cells, particularly lung cancer, telomerase is reactivated to extend the telomere and hence, tumor cells achieve proliferation [1]–[6]. Atherosclerosis is one of the key causes of coronary heart disease, and VSMC proliferation is a typical physiopathological characteristic of atherosclerosis [22], [25]. Research has shown high telomerase activity in neutrophils from unstable coronary plaques [26]. In this study, our data show that SNP of rs2736100, together with other known factors, were associated with the presence of arteriosclerosis. Our study demonstrated that the GG genotype of rs2736100 is an independent factor that is associated with the presence of arteriosclerosis (OR = 1.74 in our model). There were no significant differences in allele G frequency between arteriosclerosis patients (46.4%) and controls (43.0%), suggesting that only homozygous GG is associated with arteriosclerosis.

Of the clinical parameters, cholesterol, low-density lipoprotein and apolipoprotein B displayed collinearity, this is due to the biochemical relation between serum lipids and apolipoproteins. So we only input cholesterol into the multivariate logistic regression analysis. Cholesterol and high-density lipoprotein were demonstrated to be positively and negatively associated with arteriosclerosis which consistent with current reports [27]. Lipoprotein (a), uric acid, homocysteine, total bilirubin, γ-glutamyltransferase, and indirect bilirubin level were significantly higher in the arteriosclerosis group than the control group. However, when these variables were input into multivariate logistic regression analysis model, only homocysteine, total bilirubin, and indirect bilirubin were included in the final model. Lipoprotein (a), γ-glutamyltransferase, and uric acid were not independent factors in the final model, which may be due to the influence of other cofactors.

SNP assessments are now included in genome-wide association studies and replicate models to identify disease susceptibility loci. This methodology is currently the best choice, although it is time consuming and costly [28]. Investigation of the relationship between a certain disease and a SNP screened using molecular and cellular physiological background remains a cost-effective approach. In this study, we focused on rs2736100 based on many previous reports [11]–[16]. We demonstrated for the first time the association between GG genotype of rs2736100 and arteriosclerosis. The relative principle of the molecular function of rs2736100, regarding any possible direct and indirect biological impact of rs273100 on the presence of arteriosclerosis, needs to be investigated in future studies. Despite the limitations of a cross-sectional study, our analyses showed a exact effect of the rs2736100 GG genotype on the risk of arteriosclerosis. Our multivariate model included most of the previously reported arteriosclerosis risk factors plus the rs2736100 polymorphism. This implies that our results can be generalized to the Han Chinese population. The uncertainty of the odds ratios arising from the study design might be resolved in a large-scale population based study.
